# Combination Therapy of Atezolizumab, Bevacizumab, Carboplatin, and Paclitaxel for Metastatic Non-squamous Non-small Cell Lung Cancer With Epidermal Growth Factor Receptor (EGFR)-Tyrosine Kinase Inhibitor Resistance and EGFR Mutations

**DOI:** 10.7759/cureus.67307

**Published:** 2024-08-20

**Authors:** Hironori Kobayashi, Ayumu Otsuki, Sadakatsu Ikeda, Kei Nakashima, Yu Oyama

**Affiliations:** 1 Medical Oncology, Kameda Medical Center, Kamogawa, JPN; 2 Pulmonology, Kameda Medical Center, Kamogawa, JPN

**Keywords:** epidermal growth factor receptor, non-small cell lung cancer, driver mutations, nsclc, abcp, egfr, lung cancer, immunotherapy

## Abstract

Introduction: Atezolizumab, bevacizumab, carboplatin, and paclitaxel (ABCP) combination therapy has a potential efficacy in a specific subset of non-squamous non-small cell lung cancer (NSCLC) patients with epidermal growth factor receptor (*EGFR*) mutations following tyrosine kinase inhibitor (TKI) treatment. However, there is a dearth of investigations on the effectiveness of ABCP therapy as the primary outcome of *EGFR*-TKI use.

Methods: A single-center retrospective analysis was performed on 24 cases of stage IV *EGFR*-positive non-squamous NSCLC patients who received one or more lines of *EGFR*-TKI therapy and subsequently initiated ABCP therapy within the timeframe of April 1, 2019, to April 30, 2023. This study assessed overall survival and progression-free survival associated with ABCP therapy, further analyzing the overall survival data based on *EGFR* subgroups.

Results: The mean age of the cohort was 65 ± 9 years with 14 females (58%). The performance status (PS) was recorded as 0 in 13 out of 24 patients (54%) and 1 in 11 out of 24 patients (46%). Thirteen (54%) patients had a history of smoking. Adenocarcinoma histology was prevalent in all cases. The *EGFR* mutations included Ex19del in 14 patients (58%) and L858R in 10 (42%) patients. At ABCP therapy initiation, liver metastases were evident in three cases (13%) and brain metastases in eight (33%). Programmed death ligand 1 (22C3) expression levels varied, with <1%, 1-49%, and ≥50% observed in five, 11, and five cases, respectively, while data were missing for three cases. The median follow-up duration was 14.1 months, with median overall survival estimated at 23.6 months (95% CI: 14.5 months - not reached) and median progression-free survival at 5.6 months (95% CI: 4.9-11.5 months). The *EGFR* L858R mutation showed a favorable trend in overall survival compared with the *EGFR* Ex19del mutation (not evaluated vs. 23.6 months).

Conclusions: ABCP therapy for *EGFR*-positive non-squamous NSCLC is a promising option, similar to immune checkpoint inhibitor-free platinum-based combination therapy. Therefore, prospective trials are necessary to confirm the efficacy of these treatments.

## Introduction

Epidermal growth factor receptor (*EGFR*) mutations are prevalent in >50% of non-small cell lung cancer (NSCLC) cases with adenocarcinoma in Asia [1]. In cases of NSCLC with common *EGFR* mutations such as Ex19del and L858R, *EGFR*-tyrosine kinase inhibitors (TKIs) such as osimertinib play a pivotal role in the treatment regimens for palliative chemotherapy [2,3]. However, the optimal chemotherapy choice for *EGFR*-TKI treatment remains unclear. Despite the limited efficacy of immune checkpoint inhibitor (ICI) monotherapy for *EGFR*-mutant NSCLC [4-6], ongoing discussions have explored the potential advantages of incorporating ICIs into platinum-based combination therapies following *EGFR*-TKI resistance.

In a subgroup analysis of the IMpower150 trial, it was observed that combining atezolizumab with bevacizumab, carboplatin, and paclitaxel (BCP) yielded superior outcomes in terms of progression-free survival (PFS) and objective response rate (ORR) compared to BCP alone in non-squamous NSCLC patients with *EGFR* mutations or *ALK* rearrangements resistant to TKIs [7,8]. However, despite this evidence, there is a paucity of data specifically addressing the effectiveness of atezolizumab, bevacizumab, carboplatin, and paclitaxel (ABCP) therapy after *EGFR*-TKI use as the primary outcome [9-11]. Consequently, the clinical implications of administering the ABCP regimen after *EGFR*-TKI therapy remain controversial in patients with *EGFR*-mutant metastatic non-squamous NSCLC.

This study aimed to elucidate the efficacy of ABCP therapy, with an emphasis on this specific cohort. Additionally, an assessment of the effectiveness stratified by the *EGFR* subtype was also envisaged.

## Materials and methods

Study design and patient population

This was a retrospective cohort investigation conducted at a single medical institution, focusing on individuals diagnosed with stage IV non-squamous NSCLC harboring *EGFR* mutations at Kameda Medical Center. *EGFR* mutations were detected using commercially available methods approved in Japan. The inclusion criteria were patients who underwent one or more courses of *EGFR*-TKI therapy and subsequently received ABCP therapy between April 1, 2019, and April 30, 2023. This study was approved by the Institutional Review Board of Kameda Medical Center (IRB #: 22-089-230602). The requirement for written informed consent from individual patients was waived with an opt-out provision.

Treatment

The ABCP regimen was administered over four or six 21-day cycles, with the specific cycle count determined by the attending physicians. Treatment was initiated on day one of each cycle. Atezolizumab was dosed at 1,200 mg, bevacizumab at 15 mg/kg of body weight, paclitaxel at 175 mg/m^2^ of body surface, and carboplatin at an area under the concentration-time curve of 5 or 6 mg/mL/min [7]. Following completion of these cycles, patients continued to receive atezolizumab and bevacizumab until intolerable adverse effects or disease progression were encountered, as assessed according to the Response Evaluation Criteria in Solid Tumors (RECIST) criteria version 1.1. Continuation of atezolizumab after disease progression was permissible in the presence of clinically beneficial evidence [7].

Data collection and outcome measures

Patient characteristics and follow-up data were extracted from the hospital records. The baseline variables analyzed included age, sex, Eastern Cooperative Oncology Group performance status (ECOG PS) [12], smoking history, presence of liver and brain metastases, pathological findings, *EGFR* mutation subtype, and programmed death ligand 1 (PD-L1) expression status at the initiation of the ABCP regimen. PD-L1 expression was assessed in formalin-fixed tumor samples at a central laboratory using a commercially available PD-L1 IHC 22C3 pharmDx assay (Dako North America, Carpinteria, CA) [13]. The primary outcome measures were overall survival (OS) and PFS, which were evaluated by investigators according to RECIST version 1.1. Furthermore, OS data based on the *EGFR* subgroup were assessed.

Statistical analysis

Baseline characteristics were presented as mean and standard deviation for continuous data, while counts and proportions were used for categorical data. OS and PFS with ABCP therapy were assessed using the Kaplan-Meier method. Statistical analyses were conducted using R statistical software version 4.3.1 (R Foundation for Statistical Computing, Vienna, Austria).

## Results

Patient selection flow and baseline characteristics

Except for one case in which the ABCP regimen was administered prior to *EGFR*-TKI, a total of 24 cases were analyzed. The mean age of the cohort was 65.3 years, with 14 females (58%) (Table 1). The ECOG PS was designated as 0 in 13 cases (54%) and 1 in 11 cases (46%). Smoking history was documented in 13 patients (54%), and all 24 patients (100%) presented with adenocarcinomas. *EGFR* mutation distribution included Ex19del mutations in 14 cases (58%) and L858R mutations in 10 cases (42%). At the start of ABCP therapy, liver metastases were identified in three cases (13%), while brain metastases were observed in eight cases (33%). PD-L1 (22C3) expression levels were stratified as <1% (five cases, 21%), 1-49% (11 cases, 46%), and ≥50% (five cases, 21%), with missing data noted in three cases (13%). Patient backgrounds according to *EGFR* subgroups are listed in Supplementary Table A1 in the Appendix.

**Table 1:** Demographic and clinical characteristics of 24 patients in the study cohorts. Patient characteristics in the study cohort. Continuous variables are presented as means (standard deviations), e.g., 69.3 (7.1). Categorical variables are presented as counts (percentages), e.g., 5 (20%). Abbreviations: ABCP, atezolizumab, bevacizumab, carboplatin, and paclitaxel; ECOG, Eastern Cooperative Oncology Group; *EGFR*, epithelial growth factor receptor; PD-L1, programmed death ligand 1.

Patient characteristics	ABCP (n = 24)
Age	65.3 (9.1)
Sex (female)	14 (58.3％)
ECOG performance status	
0	13 (54.2%)
1	11 (45.8%)
Tobacco use history	
Never	11 (45.8%)
Current	1 (4.2%)
Previous	12 (50.0%)
Metastases	
Liver	3 (12.5%)
Brain	8 (33.3%)
Subtype of *EGFR* mutation	
Ex19del	14 (58.3%)
L858R	10 (41.7%)
Pathological finding	
Adenocarcinoma	24 (100%)
PD-L1 (22C3) status	
<1%	5 (20.8%)
1-49%	11 (45.8%)
≥50%	5 (20.8%)
No data	3 (12.5%)

Efficacy

**Figure 1:**
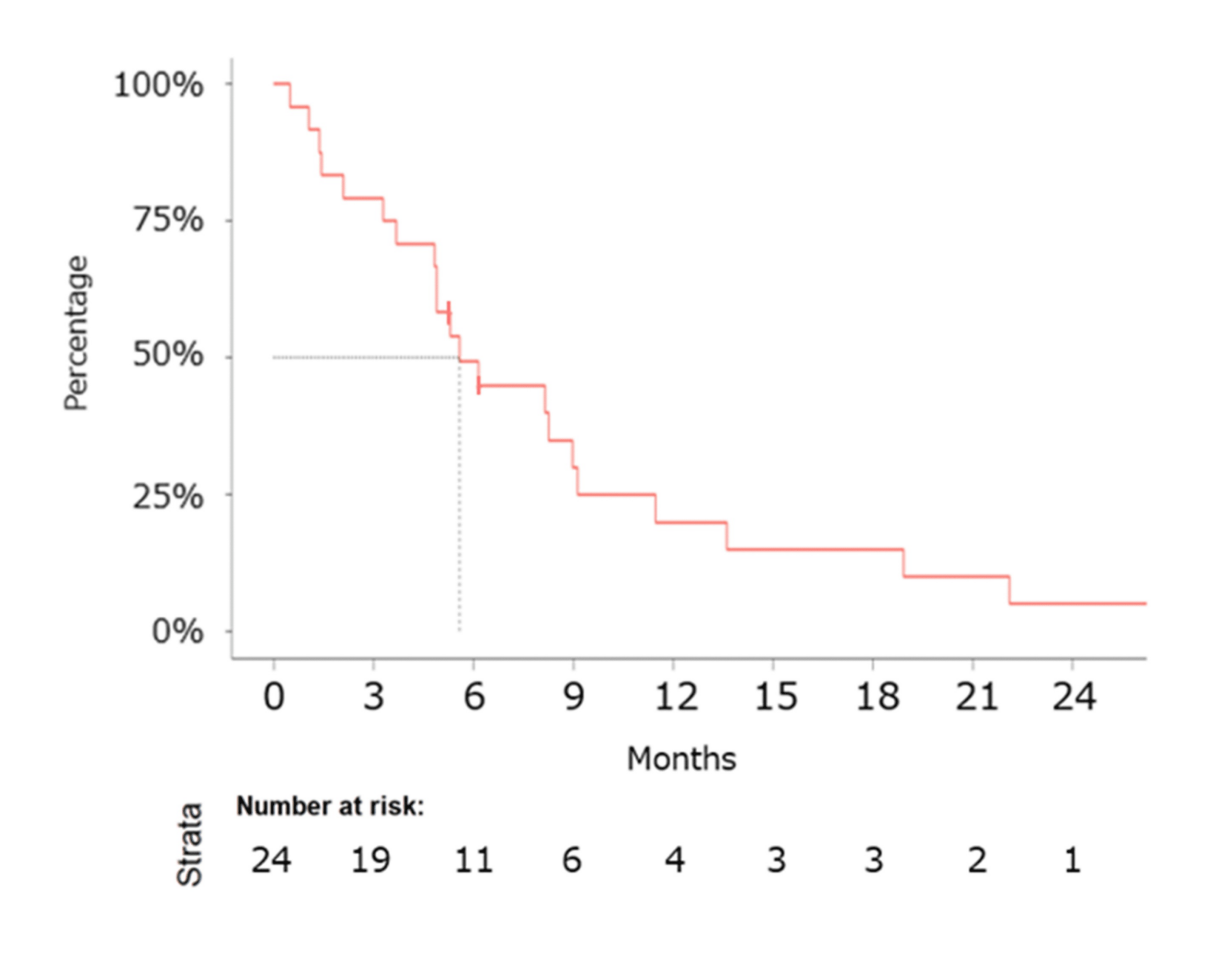
Kaplan–Meier estimates of progression-free survival. Kaplan–Meier estimates of progression-free survival (PFS) for patients treated with ABCP therapy as the primary cytotoxic regimen following EGFR-TKIs in patients diagnosed with EGFR-positive non-squamous NSCLC. The median PFS was 5.6 months (95% CI: 4.9-12 months). Abbreviations: ABCP, atezolizumab, bevacizumab, carboplatin, and paclitaxel; CI, confidence interval; EGFR, epithelial growth factor receptor; PFS, progression-free survival; TKI, tyrosine kinase inhibitor.

**Figure 2:**
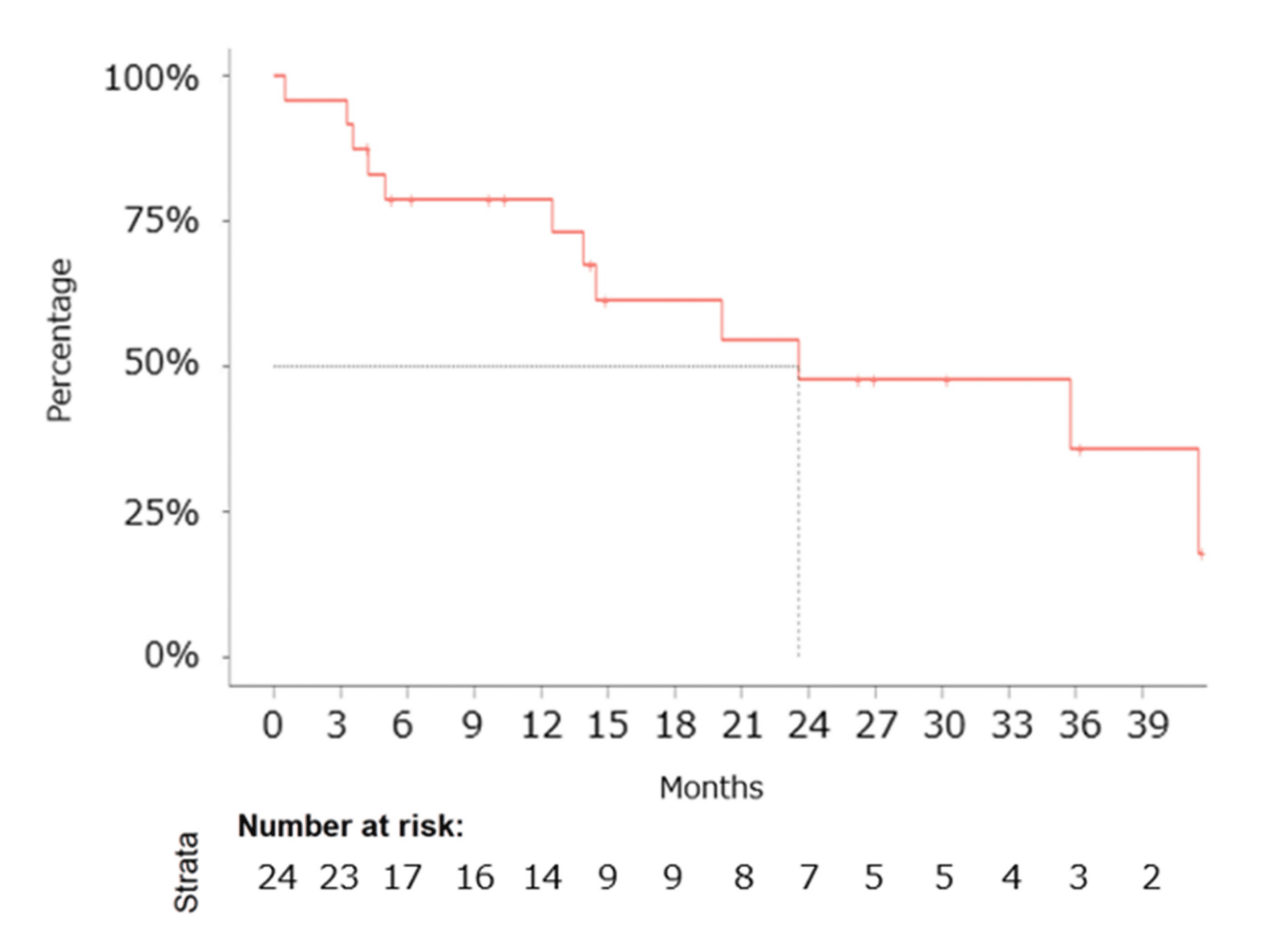
Kaplan–Meier estimates of overall survival. Kaplan–Meier estimates of overall survival (OS) for patients treated with ABCP therapy as the primary cytotoxic regimen following EGFR-TKIs in patients diagnosed with EGFR-positive non-squamous NSCLC. The median OS was 23.6 months (95% CI: 14.5 months - not reached). Abbreviations: ABCP, atezolizumab, bevacizumab, carboplatin, and paclitaxel; CI, confidence interval; EGFR, epithelial growth factor receptor; OS, overall survival; TKI, tyrosine kinase inhibitor.

**Figure 3:**
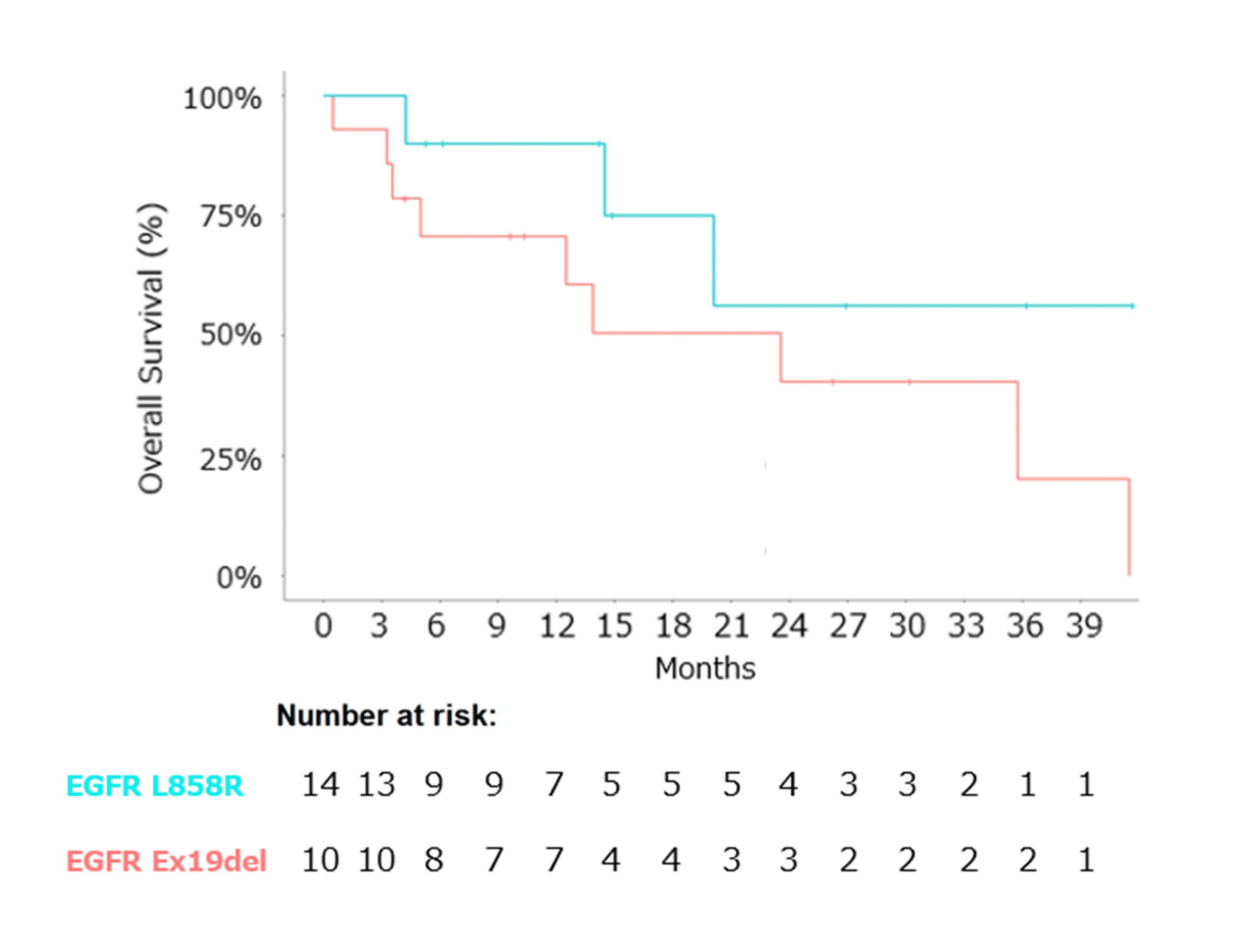
Kaplan–Meier estimates of overall survival by subtype of EGFR mutation. Caption: Kaplan–Meier estimates of overall survival (OS) by subtype of EGFR mutation. The Kaplan-Meier curve for the L858R mutation (light blue) showed a median OS of not evaluated (NE) (95% CI: 14.5 months - not reached), while that for the Ex19del mutation (light red) showed a median OS of 23.6 months (95% CI: 12.5 months - not reached). Abbreviations: ABCP, atezolizumab, bevacizumab, carboplatin, and paclitaxel; CI, confidence interval; EGFR, epithelial growth factor receptor; OS, overall survival; TKI, tyrosine kinase inhibitor.

The median follow-up duration was 14.1 months. Median PFS was determined to be 5.6 months (95% CI: 4.9-11.5 months) (Figure 1), and median OS was calculated at 23.6 months (95% CI: 14.5 months - not reached) (Figure 2). Notably, the *EGFR* L858R mutation exhibited a favorable trend in OS compared to the *EGFR* Ex19del mutation (not evaluated (NE) vs. 23.6 months) (Figure 3).

Safety

Among the 24 patients included in the study, treatment was discontinued in four patients (16.7%) due to intolerable adverse events. Specifically, two patients experienced neutropenic fever, one patient experienced anorexia, and one patient experienced pulmonary embolism.

## Discussion

In contemporary clinical practice, *EGFR*-TKIs such as osimertinib have been used as highly effective first-line chemotherapy for patients with NSCLC harboring sensitizing *EGFR* mutations (Ex19del or L858R) [2,3]. However, the choice of molecularly targeted therapies after *EGFR*-TKI administration is primarily limited to participation in clinical trials, except in instances where osimertinib-naïve patients exhibit the *EGFR* T790M mutation [14]. In patients lacking this mutation, cytotoxic chemotherapy and/or immunotherapy are viable second-line therapeutic options. Immunotherapy as a single-agent treatment yields suboptimal outcomes [4-6]. A meta-analysis demonstrated that ICIs are less effective than docetaxel when used as a second- or third-line therapy, resulting in inferior overall survival rates in NSCLC patients with *EGFR*-positive mutations [4]. Consequently, ICI monotherapy is typically reserved for later-line chemotherapy.

Conversely, the efficacy of combining ICIs with platinum-doublet chemotherapy, which is the standard first-line treatment for NSCLC lacking identifiable driver mutations, remains debatable. This study is the first to report on the efficacy of ABCP therapy in EGFR-mutant NSCLC after EGFR-TKI treatment, with a particular focus on the differences in effect between the EGFR L858R and EGFR Ex19del subtypes. Despite the absence of *EGFR*-positive NSCLC patients in most clinical trials evaluating ICIs and platinum-doublet combination chemotherapy, the IMpower150 trial uniquely included non-squamous NSCLC patients with *EGFR* mutations or *ALK* rearrangements [7,8]. Subgroup analyses from this study suggested that ABCP combination therapy is a promising and viable option for post-*EGFR*-TKI-resistant non-squamous NSCLC patients with *EGFR* mutations. Additionally, Park et al. reported a significant improvement in median PFS (8.48 vs. 5.62 months, hazard ratio (HR) = 0.62 (95% CI, 0.45-0.86); p = 0.004) and ORR (69.5% vs. 41.9%, p <0.001) among individuals with activating *EGFR* mutations or *ALK* translocations treated with ABCP compared to those treated with carboplatin and pemetrexed in the ATTLAS trial [10]. Consequently, ABCP therapy is the only validated combination regimen of ICIs and platinum-doublet chemotherapy following *EGFR*-TKI administration, as supported by findings from phase 3 clinical trials involving non-squamous NSCLC patients with *EGFR* mutations in Japan.

However, reports elucidating the efficacy of the ABCP regimen in cohorts of non-squamous NSCLC patients with *EGFR*-sensitizing mutations and resistance to *EGFR*-TKIs are scarce. In a post-hoc analysis conducted by Reck et al., an encouraging trend toward prolonged median OS was observed (NE vs. 17.5 months, HR = 0.39 (95% CI, 0.14-1.07)), alongside a statistically significant improvement in median PFS (9.7 vs. 6.1 months, HR = 0.42 (95% CI, 0.22-0.80)) when comparing ABCP with BCP. This analysis focused on a subgroup of non-squamous NSCLC patients with sensitizing *EGFR* mutations who had previously received *EGFR*-TKI treatment as part of the IMpower150 trial [9]. Furthermore, Watanabe et al. reported results from a phase 2 study (NEJ043 study), where the median PFS, OS, and ORR were 7.4 months (95% CI: 5.7-8.2), 23.1 months (95% CI: 13.1 - not reached), and 55.9%, respectively, in NSCLC patients harboring sensitizing *EGFR* mutations [11]. The median OS of 23.6 months and PFS of 5.6 months in this study were comparable to those reported previously, with the median OS exceeding 20 months following *EGFR*-TKIs, demonstrating promise in comparison to the median OS of 38.6 months and PFS of 18.9 months with osimertinib for *EGFR*-sensitizing NSCLC (FLAURA study) [2,3]. As described above, demonstrating superiority in OS, ABCP therapy has shown improved PFS and ORR in patients with *EGFR*-mutated NSCLC when administered within a selected patient group instead of ICI-free platinum-based combination regimens in daily clinical practice.

Moreover, the ABCP regimen may exhibit superior efficacy in a specific *EGFR* subtype, as the *EGFR* L858R subtype has been reported to positively influence the efficacy of ICIs in *EGFR*-sensitizing NSCLC [15,16]. This study demonstrated a more favorable trend in OS within the *EGFR* L858R mutation carrier subgroup, which is consistent with findings of monotherapy with ICIs by Takashi Ito et al., who reported that the survival of patients with L858R mutation was significantly longer than that of patients with Ex19del mutation (HR: 0.35, 95% CI: 0.13-0.93, p = 0.026) [16]. In *EGFR*-mutated NSCLC, the lower efficacy of immunotherapy can be attributed to the significantly lower tumor mutation burden (TMB) compared to that in *EGFR* wild-type NSCLC. Among them, it has been reported that *EGFR* Ex19del has a lower incidence of TMB expression than L858R [17]. Consequently, individuals with NSCLC harboring *EGFR* L858R mutation may also be better candidates for ABCP therapy, which is suggested for the first time in this study. To validate the efficacy of ABCP combination therapy in patients with *EGFR*-positive non-squamous NSCLC, especially those with *EGFR* L858R mutation, a phase 3 study with a larger cohort is warranted.

Study limitations

This study had some inherent limitations. Firstly, as a single-center retrospective study, potential concerns regarding data completeness, follow-up integrity, selection bias, and unmeasured confounding variables must be acknowledged. Despite these constraints, data collection gaps were minimal except for a detailed safety evaluation, including a comprehensive assessment of adverse events, and a satisfactory observation period was ensured, although the PD-L1 IHC 22C3 pharmDx assay was routinely employed instead of the SP142 PD-L1 immunohistochemistry assay from Ventana Medical Systems (Oro Valley, AZ). Secondly, the relatively modest cohort size increased the risk of random errors, hindering comprehensive subgroup analyses. However, the median follow-up duration of 14.1 months allowed us to observe a significant number of events, providing a robust basis for our primary endpoints, PFS and OS. While we did not perform statistical tests on subgroup outcomes due to limited event counts, descriptive analyses provided valuable insights into potential differences. Thirdly, a comparative assessment of the efficacy and safety between the ABCP regimen and the platinum doublet regimen in *EGFR*-positive (exon 19 deletion or L858R mutation) non-squamous NSCLC patients was not conducted, given the retrospective, single-arm design of this study. To address these limitations comprehensively, further randomized controlled trials involving larger cohorts are required.

## Conclusions

The use of ABCP therapy for *EGFR*-positive non-squamous NSCLC could be a promising option following *EGFR*-TKI therapy, particularly among patients with the *EGFR* L858R mutation. This study highlights the potential benefits of combining immunotherapy and chemotherapy in this specific patient group. However, validation of its efficacy through prospective cohort studies and clinical trials encompassing broader cohorts is imperative given the small sample size of this investigation. Our findings suggest that targeted combination therapies may offer improved outcomes compared to traditional chemotherapy alone, emphasizing the importance of personalized treatment strategies in oncology. Further research is needed to confirm these results and to explore the underlying mechanisms that contribute to the observed benefits.

## Appendices

Supplementary Table A1

**Table 2:** Demographic and clinical characteristics by EGFR subgroups. Patient characteristics according to *EGFR* subgroups. Continuous variables are presented as means (standard deviations), e.g., 69.3 (7.1). Categorical variables are presented as counts (percentages), e.g., 5 (20%). Abbreviations: ECOG, Eastern Cooperative Oncology Group; *EGFR*, epithelial growth factor receptor; PD-L1, programmed death-ligand 1.

Patient characteristics	Ex19del (n = 14)	L858R (n = 10)
Age	61.9 (8.9)	70.1 (7.3)
Sex (female)	7 (50.0％)	7 (70.0％)
ECOG performance status		
0	8 (57.1%)	5 (50.0%)
1	6 (42.9%)	5 (50.0%)
Tobacco use history		
Never	6 (42.9%)	5 (50.0%)
Current	1 (7.1%)	0 (0%)
Previous	7 (50.0%)	5 (50.0%)
Metastases		
Liver	2 (14.3%)	1 (10.0%)
Brain	6 (42.9%)	2 (20.0%)
Pathological finding		
Adenocarcinoma	14 (100%)	10 (100%)
PD-L1 (22C3) status		
<1%	2 (14.3%)	3 (30.0%)
1-49%	6 (42.9%)	5 (50.0%)
≥50%	4 (28.6%)	1 (10.0%)
No data	2 (14.3%)	1 (10.0%)
